# Role of angiogenic transdifferentiation in vascular recovery

**DOI:** 10.3389/fcvm.2023.1155835

**Published:** 2023-05-02

**Authors:** John P. Cooke, Li Lai

**Affiliations:** Department of Cardiovascular Sciences, Houston Methodist Research Institute, Houston, TX, United States

**Keywords:** endothelial cells, fibroblasts, nuclear reprogramming, transflammation, angiogenesis

## Abstract

Tissue repair requires the orchestration of multiple processes involving a multiplicity of cellular effectors, signaling pathways, and cell-cell communication. The regeneration of the vasculature is a critical process for tissue repair and involves angiogenesis, adult vasculogenesis, and often arteriogenesis, which processes enable recovery of perfusion to deliver oxygen and nutrients to the repair or rebuild of the tissue. Endothelial cells play a major role in angiogenesis, whereas circulating angiogenic cells (primarily of hematopoietic origin) participate in adult vasculogenesis, and monocytes/macrophages have a defining role in the vascular remodeling that is necessary for arteriogenesis. Tissue fibroblasts participate in tissue repair by proliferating and generating the extracellular matrix as the structural scaffold for tissue regeneration. Heretofore, fibroblasts were not generally believed to be involved in vascular regeneration. However, we provide new data indicating that fibroblasts may undergo angiogenic transdifferentiation, to directly expand the microvasculature. Transdifferentiation of fibroblasts to endothelial cells is initiated by inflammatory signaling which increases DNA accessibility and cellular plasticity. In the environment of under-perfused tissue, the activated fibroblasts with increased DNA accessibility can now respond to angiogenic cytokines, which provide the transcriptional direction to induce fibroblasts to become endothelial cells. Periphery artery disease (PAD) involves the dysregulation of vascular repair and inflammation. Understanding the relationship between inflammation, transdifferentiation, and vascular regeneration may lead to a new therapeutic approach to PAD.

## The current paradigm of tissue repair and vascular recovery

It is generally recognized that inflammation and inflammatory signaling plays a critical role during tissue repair. In addition, tissue repair requires restoration of tissue perfusion. As described below, these elements of tissue repair, i.e., inflammatory signaling and vascular recovery, are inextricably linked. However, before delving into the new insights linking inflammatory signaling, vascular recovery, and tissue repair, it is useful to review the current paradigm.

It is well-established that in the acute response to tissue injury, immune cells are recruited to combat bacterial infection and to clear necrotic debris. During this stage, M1-like macrophages generate inflammatory cytokines and chemokines, as well as bactericidal products such as peroxynitrite anion. This pro-inflammatory stage gives way to a regenerative phase typified by the polarization of macrophages to the M2 phenotype, which macrophage subset generates anti-inflammatory factors such as arginase, IL-10 and TGFb. At this point, the inflammation begins to subside, while tissue fibroblasts that have migrated into the injury site begin to proliferate and generate extracellular matrix proteins. Concurrently, tissue perfusion is restored, through the processes of angiogenesis and adult vasculogenesis. In addition, if the tissue injury is due to ischemia secondary to occlusive disease of larger conduit arteries, then arteriogenesis is activated to restore perfusion through the growth of collateral channels. The processes of angiogenesis, adult vasculogenesis and arteriogenesis are described elsewhere in this special issue, so we will focus on the mechanisms of angiogenic transdifferentiation, a process we first described several years ago. This process involves the transdifferentiation of a subset of tissue fibroblasts into endothelial cells, triggered by inflammatory signaling and angiogenic factors, which contributes to angiogenesis and restoration of tissue perfusion. Before launching into this discussion, lets review prior knowledge regarding the role of fibroblasts and endothelial cells in tissue repair and response to ischemia.

## Fibroblasts and endothelial cells in tissue repair and response to ischemia

Tissue repair and response to ischemia involves well-orchestrated teamwork among different cellular effectors and encompasses precisely controlled sequential events. With respect to vascular regeneration in the injured or ischemic tissue, although we appreciate that immune cells, pericytes, and smooth muscle cells are also major contributors to vasculature homeostasis and repair, we will focus on the role of fibroblasts and endothelial cells in this review, because we have discovered an intriguing link between these cell subsets in tissue repair and response to ischemia.

### Fibroblasts

There is increasing evidence of heterogeneity of tissue fibroblasts. Furthermore, the response of specific subsets of fibroblasts to injury or ischemia can determine whether the tissue repair occurs by scarring or regeneration (1–7). With genetic lineage tracing studies and single-cell sequencing, functionally distinct subsets of fibroblasts have been identified. For example, a subset of Engrailed-1(En1) lineage-positive fibroblasts are reported to contribute to scar formation in the skin after injury (8), whereas En-1 negative fibroblasts proote skin regeneration without scarring (9). Also, subpopulations of cardiac fibroblasts that express high levels of collagen triple helix repeat containing 1 (Cthrc1) (10) or Thbs4 (11) emerge after myocardial infarction to promote a profibrotic response. The presence of pro-fibrotic or regenerative fibroblasts is also dependent upon the tissue site and context as well as the developmental stage. Identifying bona fide markers for regenerative or fibrotic fibroblasts, and the molecular pathways that promote these different phenotypes is of great interest in regenerative medicine.

Inflammatory mediators modulate the fibroblast response to injury in the skin, heart, brain, and other organs (12–15). A recent study of fibroblasts from reindeer skin shed light on different fibroblast subsets and their interplay with immune cells. Fibroblasts derived from the skin (velvet) overlying the antlers suppress immune cell infiltration and support cutaneous regeneration. By contrast, fibroblasts derived from the skin on the dorsal surface of the animal are pro-inflammatory, intensify myeloid infiltration, and promote fibrosis (16). Similarly, in response to myocardial infarction, a subset of cardiac fibroblasts remain pathologically activated, generating pro-inflammatory signals and excessive extracellular matrix deposition, causing cardiac fibrosis and impaired ventricular function (14, 17). The activated fibroblasts express FAP (fibroblast activation protein) on their surface. Selective removal of this fibroblast subset can reduce cardiac fibrosis. Specifically, a chimeric antigen receptor (CAR) T cell therapy has recently been leveraged as a novel approach to mitigate cardiac fibrosis (18). In this work, the authors developed an immunotherapy strategy to reprogram CD5+ T cells *in vivo* with modified messenger RNA encoding a chimeric antigen receptor against FAP. The CAR T cell therapy reduced the number of FAP-expressing fibroblasts, attenuated cardiac fibrosis, and improved cardiac function in a murine model of heart failure. To conclude, different subsets of fibroblasts promote true tissue regeneration whereas others promote fibrosis.

### Endothelial cells

Endothelial cells (ECs) form the luminal lining of all blood vessels, participating in nutrient exchange (19, 20), immune cell trafficking (21, 22), and mechanotransduction (23, 24). They are also the major contributor to vascular recovery with ischemia or injury (25). Endothelial cells participate in three major processes involved in vascular regeneration: adult vasculogenesis, angiogenesis, and arteriogenesis. These processes are discussed elsewhere in this collection, so they will be only briefly mentioned here.

Vasculogenesis refers to the *de novo* generation of endothelial cells differentiated from progenitor cells in the mesoderm, which cells participate in the formation of the vasculature during early embryonic development (26). By contrast, adult vasculogenesis refers to a post-natal process by which angiogenic cells are mobilized from the bone marrow, in response to HIF-1*α* regulated factors generated by ischemic tissue. The bone-marrow-derived angiogenic cells circulate in the blood, homing to the ischemic or injured tissue, and participate in the restoration of the microvasculature. These circulating cells include myeloid angiogenic cells (which can home to the ischemic tissue, and generate angiogenic cytokines and chemokines), as well as true endothelial colony-forming cells, which can incorporate into the newly forming vasculature (27). In a mouse wound healing model bone marrow (BM)–derived endothelial progenitor cells responded to the gradient of hypoxia and were preferentially recruited to the most ischemic region to contribute to the micro-vasculature (28). Similar findings were found in muscle, heart, and other tissues (29–31). Some circulating cells that contribute to adult vasculogenesis may be derived from sites other than the bone marrow, likely the endothelium of other organs (31). In this case, it is likely that endothelial cells are mobilized from the vascular lumen into the circulation by systemic angiogenic factors, home to the ischemic site, and incorporate into the microcirculation.

Angiogenesis is the process of expansion of the microvasculature, where new microvessels form from pre-existing ones (32). Angiogenesis begins with sprouting of capillary endothelial cells into the tissue matrix, proliferation, migration, and luminogenesis to form functional microvessels. Angiogenesis is a pre-requisite for tissue regeneration and remodeling, but may also contribute to pathology. Tumor angiogenesis supports the growth and metastasis of tumors, and pathological neovascularization participates in arteriosclerosis, diabetic retinopathy, and arthritis (33). By contrast, deficient angiogenesis contributes to impaired tissue healing, as seen with chronic ischemia of peripheral arterial disease.

Whereas vasculogenesis and angiogenesis contribute to the restoration of the microvasculature, arteriogenesis is in response to large vessel disease. For example, in patients with an occluded superficial femoral artery, angiography frequently reveals collateral channels that connect branches of the deep femoral artery (which normally supplies the thigh) to branches of the popliteal artery, thus restoring flow to the lower leg. These collateral channels have a characteristic corkscrew appearance, and likely form from small pre-existing high resistance vessels. When the conduit vessels are not diseased, blood flows preferentially through them and not the collateral channels. As the conduit vessels becomes progressively narrowed with the progression of PAD, resistance to flow increases, and some flow now occurs through the collateral channels. The endothelium of the collateral channels release vasodilator factors to functionally increase vessel diameter, and generate adhesion molecules and chemokines that attract macrophages (34). Macrophage infiltration and generation of matrix metalloproteinases cause structural remodeling and enlargement of the collateral channels to complete the process of arteriogenesis (34).

Regeneration of the vasculature requires transcriptional signaling that directs the specialization of arterial, venous, lymphatic and capillary endothelial cells (35). VEGF (vascular endothelial growth factor) and Notch signaling are critical signals for arterial phenotype (36–39), whereas COUP-TFII (COUP transcription factor 2) and BMP (bone morphogenetic protein)-SMAD signaling are required for venous specification (40–43). Altered expression of these factors during development and in the post-natal state can lead to arteriovenous malformations(44, 45). Single cell RNAseq has revealed many subsets of endothelial cells, confirming the heterogeneity of this cell type (46–54). For example, in the cerebrovascular endothelium, ECs are specialized to form tight junctions that provide a relatively impermeable lining. Experimental modification of the brain-barrier, such as that mediated by ANGPT2 (angiopoietin 2) deficiency, causes a disruption of the tight junctions of brain endothelial cells, with vascular leakage (55, 56). Increased permeability of the brain endothelium may contribute to impaired cognition, as in SARS CoV 2 infection. By contrast the fenestrated hepatic endothelium provides for high permeability to support the filtration and metabolic functions of the liver. The transcriptional factor c-Maf plays a key role in the development of increased EC permeability (57). The heterogeneity of ECs provides for a microvasculature that supports specialized tissue functions. Furthermore, in response to ischemia or injury, the endothelium exhibits great plasticity, and assumes new functions to support tissue and vascular regeneration (58–61).

## Angiogenic transdifferentiation: A newly described process in vascular regeneration

### A novel process in vascular regeneration

We have discovered a novel form of vascular regeneration in our murine model of peripheral artery disease. Angiogenic transdifferentiation is a form of mesenchyme-to-endothelial transition that occurs in the setting of limb ischemia. In the setting of limb ischemia, we have observed that a subset of fibroblasts transdifferentiates into endothelial cells that incorporate into the microvasculature. Another set of fibroblasts generates angiogenic cytokines to support the process. Furthermore, this process requires both inflammatory signaling and angiogenic cytokines. The inflammatory signaling within a somatic cell triggers epigenetic changes that favor DNA accessibility, in a process that we have termed “transflammation”. In this state, the chromatin is in a more open configuration, and permissive to changes in cell fate. Cell fate transitions are then determined by the cellular milieu, e.g., angiogenic cytokines in the setting of hypoxia, which can promote mesenchyme-to-endothelial transition.

### The role of transflammation in cell fate transitions

Our discovery of transflammation owes much to the work of Shinyi Yamanaka. In 2006, he electrified the field of stem cell biology with his generation of induced pluripotent stem cells (iPSCs) (62), which work garnered the Nobel Prize in 2012. The iPSCs facilitated studies of the determinants of pluripotency; elucidation of the development and differentiation of different progenitor and somatic cell types; a greater understanding of the mechanisms of disease; and a potentially unlimited resource for cellular therapies (63, 64). The forced expression of the 4 transcriptional factors Oct3/4, Sox2, Klf4, and c-Myc (OSKM) using a retroviral vector was highly effective in generating iPSCs and widely used (65). However, to avoid the off-target effects of a strategy that integrated these exogenous genes into the human genome, we attempted to generate iPSCs using OSKM in the form of cell-permeant peptides (CPPs). This approach failed after many attempts to refine the dosing and timing, with poor induction of genes downstream of OSKM and little or no generation of iPSCs. Why were OSKM ineffective as cell-permeant proteins when a viral vector encoding OSKM was effective at generating iPSCs? Previous work by our group had shown that viral vectors could alter endothelial cell phenotype (66). Was it possible that the viral vector itself could be playing a role in a more dramatic change in cell phenotype, i.e., induction of pluripotency? To answer this question, we added a retroviral vector encoding the green fluorescent protein (GFP) together with the transcriptional factors. With some amazement, we noted that when the CPPs were combined with a retroviral vector encoding GFP, they were as effective at inducing the downstream transcriptional program as retroviral vectors encoding the pluripotency factors (67). This observation indicated that the retroviral vector itself, and not the Yamanaka factors alone, contributed to reprogramming. We subsequently showed that inflammatory activation of a somatic cell (in this case by the retroviral vector) shifted the balance of epigenetic factors to promote an open chromatin configuration. In an open chromatin configuration, OSKM could gain greater access to the chromatin to activate downstream genes. For example, histone acetyltransferase family members were upregulated, and histone deacetylase family members were reduced in their expression. These changes were associated with a gain of activating histone marks, and a loss of repressive marks, on downstream pluripotency genes. In gain- and loss-of-function studies, we found that the toll-like receptor 3 (TLR3) pathway which senses double-stranded viral RNA, was required for efficient induction of pluripotency by retroviral vectors encoding OSKM (67). Other pattern recognition receptors (e.g., RIG-I) which are stimulated by viral RNA, also involved in the induction of pluripotency by retroviral vectors encoding OSKM (68). In fact, it seems likely that any activation of inflammatory signaling within a somatic cell is likely to promote DNA accessibility and phenotypic fluidity because the knockdown of NFkB or IRF3 each reduced the generation of iPSCs (67).

Because inflammatory signaling is associated with the generation of reactive oxygen species (ROS) signaling, we were interested to understand if there was a role of ROS signaling in iPSC generation. We examined ROS production during the reprogramming of mouse embryonic fibroblasts (MEFs) employing a doxycycline-inducible cassette encoding OSKM (69). With induction of reprogramming, ROS generation increased. Scavenging the ROS with antioxidants reduced the generation of iPSCs. Pharmacological antagonists of NADPH oxidase (the major generator of ROS in fibroblasts), or genetic knockdown of the enzyme substantially decreased reprogramming efficiency. On the other hand, when we increased ROS generation substantially using genetic and pharmacological approaches the generation of iPSCs was also impaired (69). These studies indicate that there is a Goldilock's zone for inflammatory signaling and generation of ROS, where cell fate transitions are facilitated.

The activation of TLR3 also increases the expression of inducible nitric oxide synthase (iNOS), which produces nitrogen oxide radicals that can S-nitrosylate proteins to modify their activity. During transdifferentiation of fibroblasts to endothelial cells, we have observed that iNOS expression is increased in both the cytoplasm and nucleus, indicating that the enzyme translocates to the nucleus. There it binds RING1a of the polycomb repressive complex 1,and S-nitrosylates RING1a. RING1a subsequently dissociates from the chromatin, with a concomitant reduction in its suppressive histone marks (70). In addition, iNOS binds to, and S-nitrosylates, a component of the NURD complex (a deacetylase), thereby reducing histone deacetylation (71). These effects of inflammatory signaling, e.g., iNOS translocation to the nucleus and S-nitrosylation (and inhibition) of repressive epigenetic modifiers, promotes an open chromatin configuration that facilitates cell fate transitions and tissue regeneration (70, 71). Transflammation is equally critical for the induction of pluripotency, as well as the transdifferentiation of a somatic cell into another cell type (67–74).

With respect to transdifferentiation, we have been able to generate induced endothelial cells (iECs) from human fibroblasts simply by activating inflammatory signaling (with the TLR3 agonist poly inosinic:cytidylic acid, i.e., Poly I:C), and adding to the medium a cocktail of endothelial differentiating factors (i.e., VEGF, BMP4, FGF, and 8Br-cAMP, together with a TGF *β* inhibitor) (73). In the absence of Poly I:C, the endothelial differentiating factors do not induce fibroblasts to become iECs. However, with activation of inflammatory signaling, the increase in DNA accessibility facilitates the transcriptional direction provided by the endothelial differentiation factors, and iECs are generated.

### Role of transflammation in the response to injury

**Figure 1:**
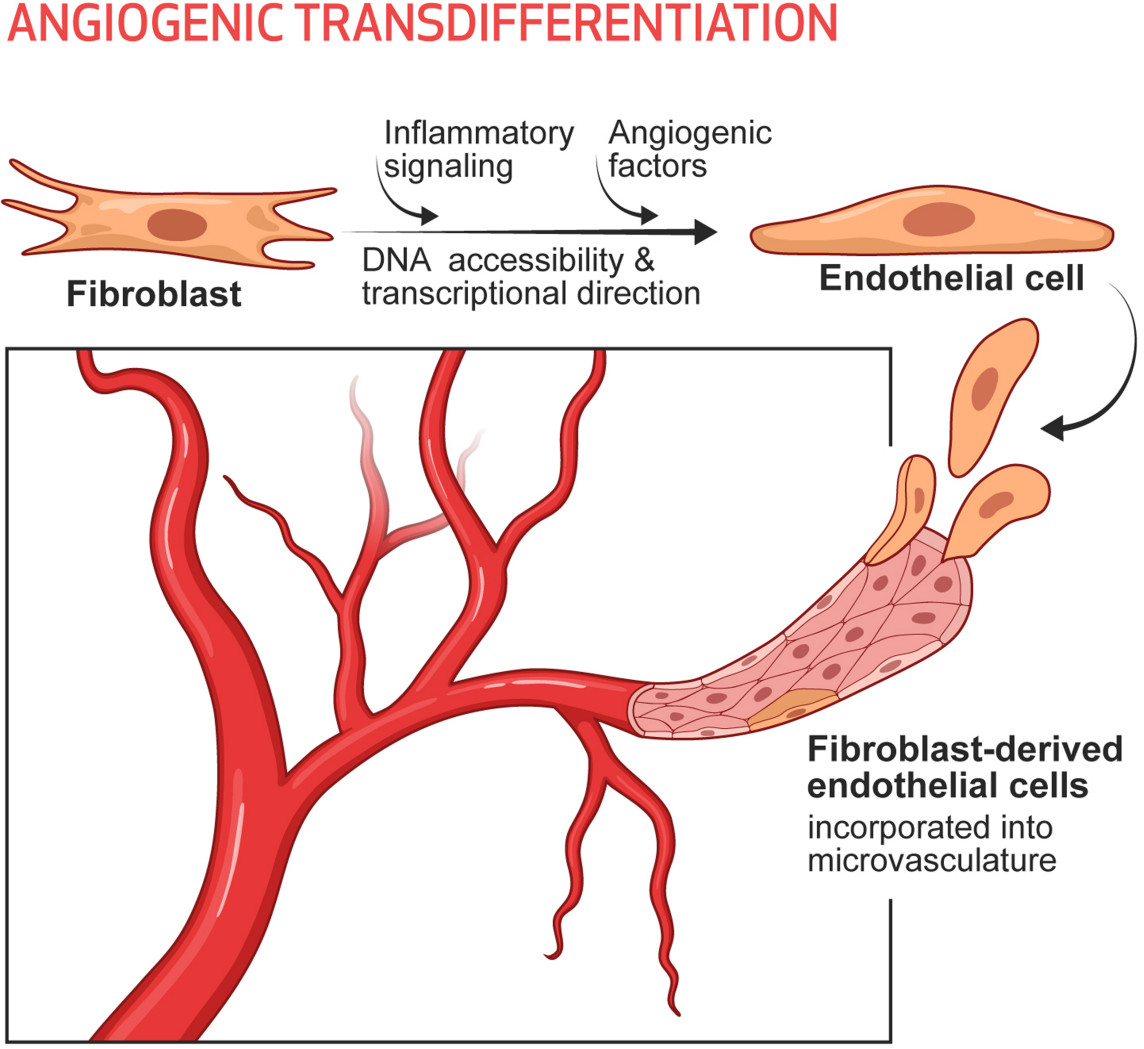
Angiogenic transdifferentiation contributes to the expansion of the microvasculature, particularly in the setting of ischemia. Under the influence of inflammatory signaling (which increases DNA accessibility) and environmental cues (such as angiogenic cytokines, which provide transcriptional direction) some subset(s) of fibroblasts can transdifferentiate into endothelial cells. These induced endothelial cells contribute to the expansion of the microvasculature as a response to ischemia and augment tissue perfusion.

It seems likely that in the setting of tissue injury or ischemia, the subsequent inflammatory signaling increases DNA accessibility in the cells of the damaged tissue. In our conceptual model the increased DNA accessibility would promote cellular plasticity, and adaptive changes in cell phenotype. Indeed, we have tested this hypothesis in the murine model of limb ischemia, using single cell RNA sequencing and cell lineage tracing (74). In this model, we were able to define 8 subsets of fibroblasts in the mouse hindlimb, each of which subsets were detected by lineage tracing and expressed a set of typical fibroblast genes such as Col1a. Two of these fibroblast subsets increased in the ischemic limb. One subset (cluster 5) expressed some typical endothelial identity genes, whereas another (cluster 8) expressed some angiogenic cytokines. Using FACS sorting and antibodies to surface markers for these clusters, we pulled out cells that resembled fibroblasts in 2D culture. However, when we added the cells to Matrigel, cluster 5 cells formed a network very similar to that formed by genuine endothelial cells and expressed endothelial surface markers, whereas cluster 8 continued to morphologically resemble fibroblasts (which form nodules in Matrigel) but made angiogenic cytokines. The importance of inflammation in the generation of these clusters, and their contribution to perfusion, was demonstrated by pharmacological (dexamethasone) or genetic knockdown (p65 kd) of inflammatory activation. Pharmacological or genetic suppression of inflammatory activation markedly reduced the numbers of iECs detected (by lineage tracing), limited recovery of perfusion (by laser doppler spectroscopy), and impaired healing of the hindlimb (74). To conclude, a subset of fibroblasts in the hindlimb appears to be poised to become endothelial cells under the influence of inflammatory activation and angiogenic factors generated during hypoxia. Furthermore, these endogenous iECs appear to contribute significantly to the restoration of perfusion and tissue healing. We call this process Angiogenic Transdifferentiation (74) (Figure 1).

## The role of metabolism in transdifferentiation

Metabolic regulation of DNA accessibility has emerged as a key regulatory process controlling cell fate (75). It has been intensively investigated regarding the bidirectional regulation between immune cells and metabolism (76–79). However, less is known about the role of cell-autonomous immune signaling on non-immune cells responding to tissue damage (79). We have recently shown that metabolic modulation of epigenetic regulation plays an important role in cell fate decisions in non-immune cells (80). In our study, we found that innate immune activation triggers a glycolytic shift that is required for transdifferentiation. In parallel, the mitochondrial citrate transporter (Slc25a1) is upregulated to facilitate the export of citrate out of the mitochondria. Concurrently, there is an increase in the expression of nuclear ATP citrate lyase (ACL), which converts citrate to acetyl-CoA to support the increase in histone acetyltransferase activity. The subsequent increase in histone acetylation increases DNA accessibility and facilitates the transdifferentiation of fibroblasts to endothelial cells. This work indicates an important role for metabolism during transdifferentiation and vascular regeneration.

## A potential therapeutic avenue for PAD

Ischemic cardiovascular disease is the leading cause of disability, morbidity, and mortality globally. Of concern, ischemic vascular disorders, such as myocardial infarction, cerebrovascular disease, peripheral vascular disease, and microcirculatory disorder, are increasing worldwide (81). Peripheral artery disease(PAD) occurs in about 12% of the U.S. adult population over the age of 60 (82), and in the most severe form of critical limb ischemia (CLI), is associated with a mortality rate of about 20% within 1 year of diagnosis. Endovascular procedures and surgical bypass for CLI are useful in salvaging limbs but amputation-free survival in the absence of efficacious medical treatment for limb ischemia remains poor (83). Although angiogenesis is critical for perfusion recovery (84), angiogenic therapies have largely failed in improving function (e.g., walking distance) and in alleviating complications (e.g., ischemic ulcers) of CLI (85, 86). We hypothesize that a reduction in DNA accessibility and cellular plasticity may be playing a significant role in the impairment of angiogenesis and arteriogenesis that is observed in CLI patients. Because these patients have evidence of significant inflammation, particularly in the region of ischemia and non-healing ulcers, it is likely that in these regions, inflammatory signaling is excessively activated. As we have shown, a modest amount of inflammatory signaling is necessary for DNA accessibility and cell fate transitions. However, in the face of excessive inflammatory activation, cellular plasticity is impaired (69, 71). Recent clinical trials using anti-inflammation strategies showed promising results in lowering the risk of major adverse cardiovascular events (87–89). Excessive inflammatory signaling is clearly involved in the progression of cardiovascular disease (90–94), and it may also be involved in the impairment of tissue healing that is frequently associated with cardiovascular disease. However, because an optimal amount of inflammatory signaling is also necessary for angiogenic transdifferentiation and wound healing, we feel that caution should be exercised in the use of these agents in patients with healing wounds or tissue damage (95). It would be of great clinical merit to define the optimal range of inflammatory signaling during the healing stages; develop diagnostics that could quantify the degree of inflammatory signaling in the target tissue; and therapeutics that could modulate the level of inflammatory signaling.

In this regard, there is a deficiency of multi-omics information for the affected tissues in PAD. It will be useful to characterize the cell composition, and the genetic, epigenetic, and proteomic features of each cell subtype in patient samples vs. those from healthy individuals; from patients with PAD vs. those with CLI; and from those individuals that do not have PAD but have the pre-requisite risk factors for PAD. Such studies might identify deficiencies or aberrations in cell subtypes that play a role in the disease and might identify pathways that are contributory. Such information could provide a greater understanding of the heterogeneity in the presentation of PAD; could lead to mechanistic insights into etiology; and could provide avenues toward novel therapies.

## Conclusion

Vascular regeneration in the adult includes the processes of angiogenesis, arteriogenesis, and adult vasculogenesis. To these, we add the new process of angiogenic transdifferentiation. Angiogenic transdifferentiation involves a cell fate switch that requires an optimal amount of inflammatory signaling (which increases DNA accessibility) and angiogenic factors (that provide transcriptional direction). This newly discovered response to ischemia appears to play an important role in the expansion of the microvasculature and the restoration of perfusion.
